# HTD2: a single-crystal X-ray diffractometer for combined high-pressure/low-temperature experiments at laboratory scale

**DOI:** 10.1107/S160057672200766X

**Published:** 2022-09-28

**Authors:** Andreas Fischer, Jan Langmann, Marcel Vöst, Georg Eickerling, Wolfgang Scherer

**Affiliations:** a CPM, Institut für Physik, Universität Augsburg, 86159 Augsburg, Germany; HPSTAR and Harbin Institute of Technology, People’s Republic of China

**Keywords:** HTD2, low temperature, high pressure, single crystals, instrumentation

## Abstract

High-pressure (HP) X-ray diffraction experiments at low temperature (LT) require dedicated instruments as well as non-standard sample environments and measuring strategies. This is especially true when helium cryogenic temperatures below 80 K are targeted. Furthermore, only experiments on single-crystalline samples provide the prerequisites to study subtle structural changes in the *p*–*T* phase diagram under extreme LT and HP conditions in greater detail. Due to special hardware requirements, such measurements are usually in the realm of synchrotron beamlines. This contribution describes the design of an LT/HP diffractometer (HTD2) to perform single-crystal X-ray diffraction experiments using a laboratory source in the temperature range 400 > *T* > 2 K while applying pressures of up to 20 GPa.

## Abstract 2

The design and operation of a newly commissioned single-crystal X-ray diffractometer (HTD2) are presented. The device enables experiments under simultaneous low-temperature and high-pressure conditions using a laboratory X-ray source.

## 1. Introduction

The quest for the observation of superconductivity at room temperature has so far culminated in hydrogen-rich materials like H_3_S (Drozdov *et al.*, 2015 ▸), LaH_10_ (Drozdov *et al.*, 2019 ▸) and a controversially discussed carbonaceous sulfur hydride with transition temperatures of up to 287.7 K at an applied pressure of 267 GPa (Snider *et al.*, 2020 ▸). High-pressure X-ray diffraction (XRD) experiments can play and already have played an important part in this quest (Drozdov *et al.*, 2019 ▸) by providing structural information on the superconducting phase to stimulate further theoretical work.

Yet, a wealth of physical phenomena with potential structural implications can only be studied by combining high-pressure (HP) with low-temperature (LT) studies. This is, for example, the case for the metal-to-insulator transitions in NiS_2_ (Feng *et al.*, 2011 ▸), FeS (Kobayashi *et al.*, 2005 ▸) and PrFe_4_P_12_ (Hidaka *et al.*, 2005 ▸). Also, the fascinating interplay between charge-density wave formation and the onset of superconductivity in low-dimensional compounds such as Ir_1−*x*_Pt_*x*_Te_2_ (Kiswandhi *et al.*, 2013 ▸), CsV_3_Sb_5_ (Yu *et al.*, 2021 ▸) and TiSe_2_ (Joe *et al.*, 2014 ▸) critically depends on both control parameters: pressure and temperature.

Although turn-key solutions for the in-laboratory measurement of physical properties under combined LT/HP conditions are readily commercially available (for example in the case of Quantum Design’s MPMS/PPMS devices equipped with pressure cells), XRD measurements under the same conditions are still an expert topic and usually limited to diffractometers at specialized synchrotron beamlines (Gerard & Pernolet, 1973 ▸; Morosin & Schirber, 1974 ▸; Chasseau *et al.*, 1993 ▸; Tang *et al.*, 1998 ▸; Ohwada *et al.*, 2001 ▸; Takemura *et al.*, 2002 ▸; Suzuki & Endo, 2003 ▸; Guionneau *et al.*, 2004 ▸; Yoshimura *et al.*, 2006 ▸; Bizen *et al.*, 2008 ▸; Zimmermann *et al.*, 2008 ▸; Mittal *et al.*, 2011 ▸; Shepherd *et al.*, 2011 ▸; Li *et al.*, 2012 ▸; Tse *et al.*, 2012 ▸; Cameron, 2014 ▸; Yamada *et al.*, 2014 ▸; Hejny & Minkov, 2015 ▸; Elatresh *et al.*, 2017 ▸; Shen & Mao, 2017 ▸; Povedano *et al.*, 2020 ▸).

**Figure 1:**
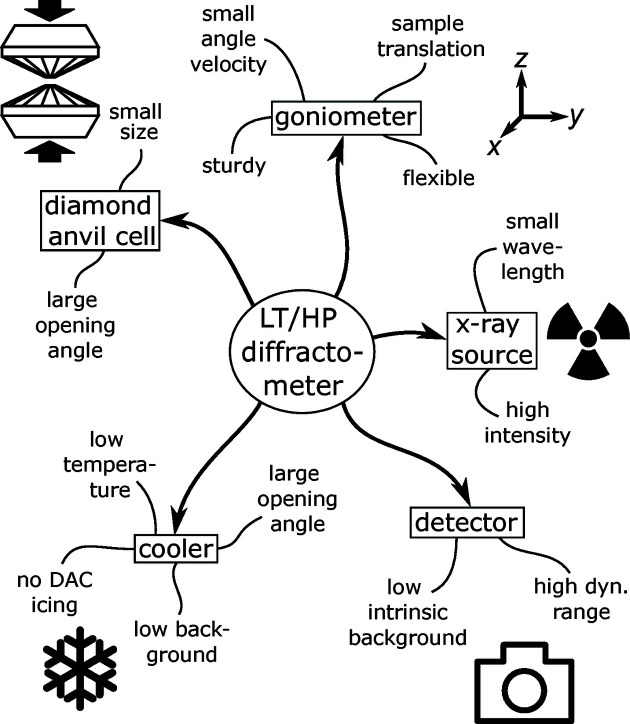
Overview of the requirements on the components of an LT/HP diffractometer.

In this contribution we describe a new instrument for in-laboratory LT/HP single-crystal XRD experiments (HTD2) built from commercially available components. We first sketch the special requirements on such an instrument (see Fig. 1 ▸). Then we proceed to describe the specific setup and choice of hardware components to meet these requirements and briefly describe the software tools to control the interaction of the individual components to measure precise XRD data. In a final step, we focus on the operation of the diffractometer under ambient and LT/HP conditions.

### 1.1. General aspects

The investigation of the pressure-dependent evolution of a crystal structure by XRD usually hinges on the use of a diamond anvil cell (DAC) (Van Valkenburg, 1964 ▸) in transmission geometry.^1^ Several experimental restrictions are an inherent consequence of the DAC design. Most prominently, the opening angle of the DAC limits the accessible portion of reciprocal space (Miletich *et al.*, 2000 ▸; Angel *et al.*, 2000 ▸; Katrusiak, 2008 ▸; McMahon, 2012 ▸; McMahon *et al.*, 2013 ▸; Hejny & Minkov, 2015 ▸). Therefore, a large opening angle can be advantageous.

Cooling of the DAC in an LT measurement can be realized by open-flow techniques featuring a cold gas stream or conduction cooling inside a closed sample environment (Gerard & Pernolet, 1973 ▸; Morosin & Schirber, 1974 ▸; Silvera & Wijngaarden, 1985 ▸; Chasseau *et al.*, 1993 ▸; Simmons *et al.*, 1993 ▸; Tang *et al.*, 1998 ▸; Ohwada *et al.*, 2001 ▸; Takemura *et al.*, 2002 ▸; Suzuki & Endo, 2003 ▸; Guionneau *et al.*, 2004 ▸; Bizen *et al.*, 2008 ▸; Zimmermann *et al.*, 2008 ▸; Mittal *et al.*, 2011 ▸; Cameron, 2014 ▸; Shen & Mao, 2017 ▸; Almax EasyLab, Diksmuide, Belgium). Both methods benefit from a compact DAC design: in the case of open-flow cooling, the DAC has to be small enough to fit into the sheath current. Otherwise, ice formation at the surface of the pressure cell severely hampers the LT/HP study by parasitic Bragg peaks. This can be overcome by employing conduction cooling when the pressure cell is placed in the evacuated sample chamber of a cryostat. In addition, the reduced heat capacity of a small DAC helps to reach LT with the limited cooling power available in a conduction cooling scenario.

**Figure 2:**
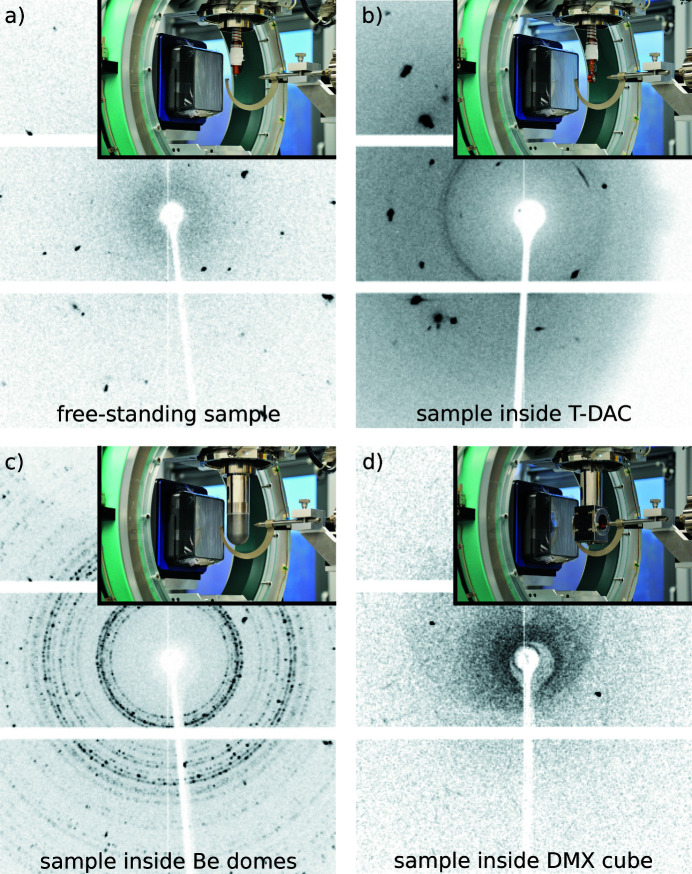
Example XRD images demonstrating the combination of sample scattering, parasitic scattering and shadowing present when working with (*a*) a free-standing Sc_3_CoC_4_ sample, and a Sc_3_CoC_4_ sample inside (*b*) a T-DAC, (*c*) beryllium domes or (*d*) a DMX cube with two Kapton windows. Photographs of the experimental setups used for collecting the example images are shown in the respective insets.

But despite its advantages for LT/HP experiments, conduction cooling also poses specific problems for XRD applications. Most importantly, the body of the required vacuum chamber usually interferes with the X-ray beam path (Macchi, 2012 ▸). This leads to additional X-ray absorption and background scattering, and in some cases also results in a further reduction of the already restricted accessible portion of reciprocal space. The severity of each of these problems can be influenced by the design and material of the vacuum chamber body: Kapton foil absorbs little X-ray radiation, generates only weak and well behaved background scattering [Fig. 2 ▸(*d*)] (Peterson & Yang, 2000 ▸; Meserschmidt *et al.*, 2003 ▸), and reflects relatively little thermal radiation. It is usually used to furnish a robust but X-ray-blocking steel body with nearly X-ray-transparent entry and exit windows [inset in Fig. 2 ▸(*d*)]. In contrast, beryllium metal as an alternative material generates stronger and more inhomogeneous background scattering than Kapton foil (Meserschmidt *et al.*, 2003 ▸; Coome *et al.*, 2012 ▸; McMonagle & Probert, 2019 ▸). However, it reflects thermal radiation more effectively and is robust enough to be manufactured into a thin and monolithic dome [inset in Fig. 2 ▸(*c*)]. Unlike a steel vacuum chamber body with Kapton windows of limited size, such a beryllium dome imposes no additional geometric limitations beyond the DAC opening angle.

A suitable diffractometer design helps to alleviate some of the restrictions due to the LT/HP equipment. Choosing an X-ray source with a short wavelength decreases the spacing between the reflection positions on the detector, so that a maximum number of reflection intensities can be measured with the given opening angles of the vacuum chamber and DAC (McMahon, 2012 ▸). This gain in the number of sampled reflections, however, comes at the cost of disadvantages like reduced X-ray scattering intensities (Angel *et al.*, 2000 ▸) or a smaller resolution in lattice plane spacings (*d*) with a given detector-to-sample distance and detector pixel size (Shen & Mao, 2017 ▸). Independent of the employed X-ray wavelength, a sturdy and flexible goniometer is advantageous to optimally sample the accessible portion of reciprocal space (Angel *et al.*, 2000 ▸).

Further requirements on the diffractometer design come from the object of study in the LT/HP experiments itself. A typical application of such an instrument is the investigation of structural phase transitions that may be indicated by only faint diffraction features like the formation of superstructure reflections. To measure weak reflections in the presence of strong reflections may require extended exposure times and a detector with a high dynamical range and a small intrinsic background, as well as a goniometer permitting scans of the main rotational axis at low speed. Even though the last aspect may be worked around by oscillating scans, the build-up of errors due to gear slippage should be kept minimal.

A further major issue in LT/HP XRD studies is getting and keeping the sample crystal centered in the X-ray beam (Peterson & Yang, 2000 ▸; Angel *et al.*, 2000 ▸; Budzianowski & Katrusiak, 2004 ▸; Dawson *et al.*, 2004 ▸; McMahon *et al.*, 2013 ▸). Temperature-cycling results in a contraction/expansion of the DAC mount and thus an altered sample position. During an LT/HP measurement, however, the DAC body and cooling equipment (*e.g.* the beryllium domes) hide the crystal position from view. This problem can be resolved with the help of a motorized sample mount. A controlled step-wise DAC translation along three orthogonal axes allows for crystal realignment by maximizing the scattered radiation intensity.

## 2. System setup

### 2.1. Hardware

**Figure 3:**
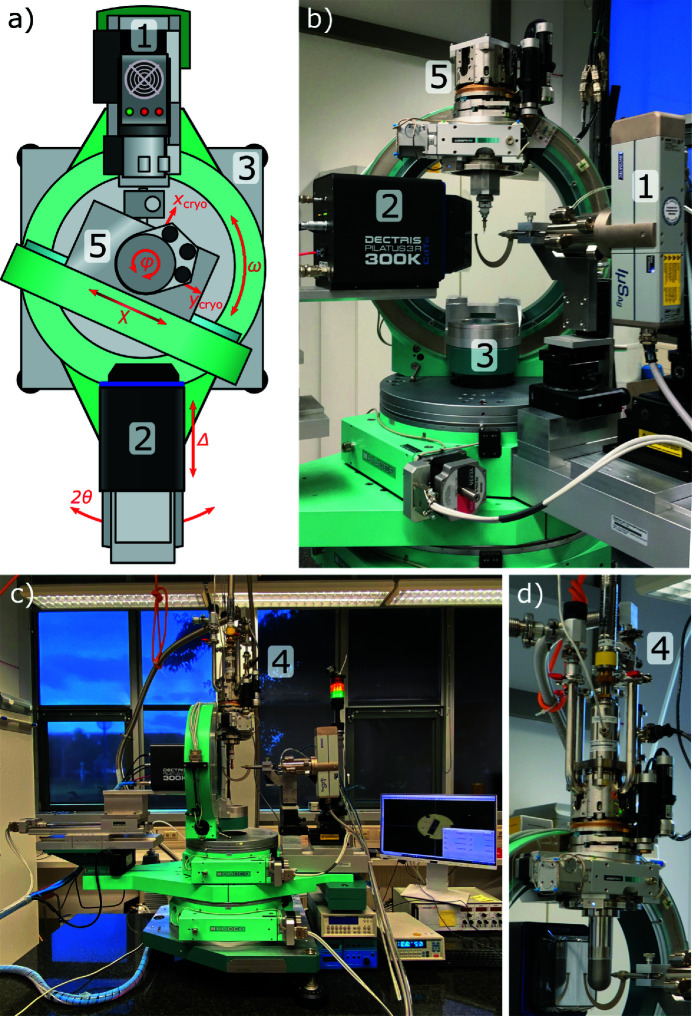
(*a*) System overview of the HTD2, and photographic images of the setups for (*b*) collecting high-resolution data under ambient conditions and (*c*) collecting LT/HP data. An enlarged view of the sample cryostat with mounted beryllium domes is given in (*d*). Numerical labels are explained in the text.

A major objective for the hardware setup of the HTD2 was the use of readily available commercial components. We therefore chose a micro-focus sealed-tube X-ray source (Incoatec Ag IµS 3.0 Upgrade; 1 in Fig. 3 ▸) equipped with focusing mirror optics (Montel-type, divergence 5 mrad, FWHM 100 µm at the sample position). Scattered intensities are measured by a hybrid photon counting detector (DECTRIS PILATUS3 R 300K; 2 in Fig. 3 ▸) with a CdTe detection layer optimized for short wavelengths (>90% quantum efficiency for Ag *K*α). The detector also provides a large dynamic range (20 bits) required for the detection of weak/diffuse scattering intensities next to strong Bragg reflections. Both detector and source are mounted on a four-circle Eulerian cradle goniometer (HUBER; 3 in Fig. 3 ▸), consisting of a 440 series 2θ circle with base, a 430 series ω circle, a model 512.1 closed χ Eulerian cradle and a model 410A-X2W1 φ circle, the last equipped with optical position encoder. The gear ratio of the scanning φ-axis step motor was chosen such as to allow for scans as slow as 15 min/0.5°. Also, a rotation of the 2θ arm passing the χ cradle is possible using the motorized detector translation stage model 5101.3 (HUBER). This enables positioning of the detector center at 2θ angles as high as 125°, which translates into a theoretical maximum data resolution close to the limiting sphere of Ag *K*α radiation.

Four options for cooling of the sample are available: (i) a standard Oxford Cryostream 700 N_2_ open-flow cooling device for temperatures 400 > *T* > 80 K, (ii) an Oxford Helijet He open-flow cooling device for temperatures 100 > *T* > 10 K, (iii) an ARS closed-cycle He Displex (DE-202SG) for temperatures 300 > *T* > 4 K and (iv) an ARS He-flow cryostat (Helitran LT-3G; 4 in Fig. 3 ▸) for temperatures 80 > *T* > 2 K. Temperatures above the base temperatures of options (iii) and (iv) can be reached by a combination of a PID temperature controller (Lake Shore 331/335), silicon diode temperature sensors (Lake Shore DT-670-SD) positioned at the cooling tip as well as close to the sample position, and a heating wire wound around the cooling tip. For an accurate positioning and centering of cryostats (iii) and (iv) the three-axis motorized mount 5106.20M (HUBER; 5 in Fig. 3 ▸) is employed. Two types of vacuum and radiation shields are available for use in cryostats (iii) and (iv). The first option is the ARS DMX cube vacuum and radiation shield [inset in Fig. 2 ▸(*d*)] which allows for visual inspection of the sample position through exchangeable windows fabricated from quartz, Kapton or KBr. As a second option for the lowest possible temperatures, we employ a set of beryllium domed vacuum and radiation shields [inset in Fig. 2 ▸(*c*)]. However, these cause parasitic background noise due to scattering from the partially crystalline beryllium metal of the domes [Fig. 2 ▸(*c*)]. A subtraction of the parasitic beryllium scattering is possible by collecting individual background images for each data frame. Therefore, we have equipped the cold tips of both He cooling devices with piezo driven nanopositioners (attocube ANPz30) that allow for an *in situ* translation of the sample into and out of the X-ray beam (for details of this procedure, see Section 3.2).

In a combined LT/HP experiment, pressures up to ∼20 GPa can be generated using a Diacell Tozer-type DAC (Graf *et al.*, 2011 ▸; Almax EasyLab, Diksmuide, Belgium) [T-DAC; Fig. 5(*b*)] featuring an opening angle of 82° and Boehler–Almax-type anvils (Boehler & De Hantsetters, 2004 ▸). The maximum achievable pressure, however, has a strong dependence on experimental parameters such as the culet diameter of the employed diamond anvils. In practice, we have generated pressures up to ∼6 GPa. The small size of the T-DAC (diameter: 12 mm, height: 11.5 mm) renders it suitable for conduction cooling to temperatures as low as 2 K using a helium-flow cryostat (iv). Good thermal coupling to the cryostat cooling tips is afforded by a specially designed copper mount [visible in Fig. 5(*b*)]. Applied pressures are determined ahead of the XRD experiments at room temperature using fluorescence measurements on ruby spheres inside the pressure chamber (Forman *et al.*, 1972 ▸; Barnett *et al.*, 1973 ▸; Piermarini *et al.*, 1975 ▸; Mao, 1978 ▸; King & Prewitt, 1980 ▸; Bell *et al.*, 1986 ▸; Mao *et al.*, 1986 ▸; Eggert *et al.*, 1989 ▸, 1991 ▸; Datchi *et al.*, 2007 ▸; Shen *et al.*, 2020 ▸).

### 2.2. Software and control

**Figure 4:**
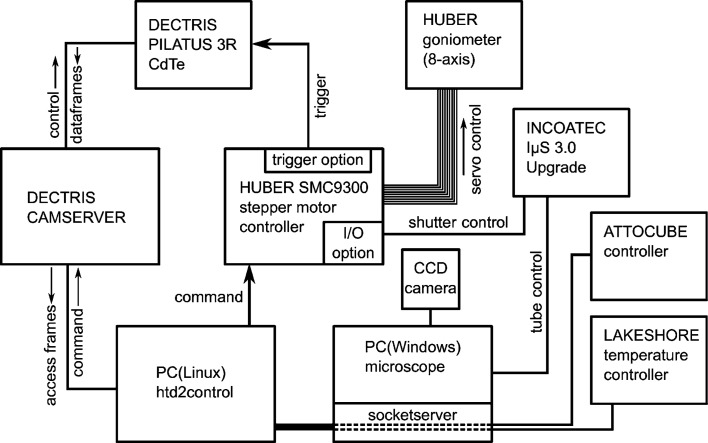
Diagram of the components and interfaces of the HTD2.

A schematic overview of the different components and their control channels is provided in Fig. 4 ▸. An eight-axis HUBER stepper motor controller (model SMC9300) represents the heart of the diffractometer and controls all stepper motors of the circles 2θ, χ, ω and φ as well as the detector translation Δ and the three cryostat-holder axes *x*_cryo_, *y*_cryo_ and *z*_cryo_ [indicated in Figs. 3 ▸(*a*) and 5 ▸]. The controller is equipped with an 8-bit digital I/O option that is connected to the Incoatec ISG250 X-ray generator and allows for the control of the safety shutter of the IµS tube. Owing to its direct X-ray detection capability the DECTRIS PILATUS3 R CdTe detector counts photons only when activated, so that a dedicated measurement shutter can be omitted. The remaining safety shutter is only opened/closed at the beginning/end of an experiment. To prevent any intensity losses during detector readout (∼7 ms), the standard mode of operation is a stepwise movement of the φ circle (using the fastest possible acceleration/deceleration ramps). This approach is of special importance in cases of small exposure times (for example, when a fine frame slicing is used). In order to keep the goniometer motion in sync with the detector readout periods, the SMC9300 is equipped with a dedicated trigger option board. It is connected to the external trigger input of the DECTRIS detector and activates the detector once the acceleration ramp is finished.

All components are controlled with the software package *HTD2CONTROL* written in Python (using event loops and multithreading). It provides an extensive *tkinter*-based graphical user interface (GUI) intended to reconcile user friendliness and maximum flexibility in using the diffractometer. *HTD2CONTROL* is run on a Linux PC (Fig. 4 ▸). It enables the collection of multiple scan sets without user intervention and features basic plausibility/safety checks of entered scan settings for crash prevention. All communication with the remote devices SMC9300, DECTRIS framegrabber and other controllers takes place via simple ethernet sockets. For local testing and development the *HTD2CONTROL* package also contains a set of software servers which emulate all hardware components.

A separate Windows PC inside the control hutch (Fig. 4 ▸) is used to display the image from the CCD camera of the optical microscope, control the SMC9300 during crystal centering operations and control the operating settings of the X-ray tube. Furthermore, the PC is connected to the Lake Shore and attocube controllers via an RS232/USB interface and makes them available to *HTD2CONTROL* via dedicated socket­servers written in Python. This setup enables the diffractometer control software to adjust and log the sample temperature during measurements using the He cryostats (iii) and (iv) and to translate the sample into and out of the X-ray beam as needed for data collection with foreground and background images (see Section 3.2).

## 3. Diffractometer operation

Before discussing the overall performance of the instrument in Sections 3.3 and 3.4, we focus on the practical aspects of overcoming the two main experimental obstacles for LT/HP experiments, namely the centering of the sample and the treatment of parasitic scattering by sample environments.

### 3.1. Crystal centering

In contrast to experiments under ambient conditions, additional sample environments in experiments under LT/HP conditions (body of a DAC, heat and radiation shields) partly or completely obscure the direct view of the sample. Still visible sample fractions may be subject to substantial parallax effects, for example from the diamond anvils (Angel *et al.*, 2000 ▸; Budzianowski & Katrusiak, 2004 ▸; Dawson *et al.*, 2004 ▸; McMahon *et al.*, 2013 ▸). These problems collectively render a routine optical centering of the crystal into the goniometer center difficult or even impossible.

Diffraction-based crystal centering provides a possible alternative, as X-rays penetrate thin beryllium walls and are not significantly refracted by diamond. But centering approaches based on reflection positions on the detector (King & Finger, 1979 ▸; Dera & Katrusiak, 1999 ▸; Probert *et al.*, 2010 ▸) are iterative and rather time consuming and require precise knowledge of the X-ray source and goniometer characteristics. Less prior information is needed for the raster-scanning approaches applied frequently at synchrotron beamlines (Karain *et al.*, 2002 ▸; Song *et al.*, 2007 ▸; Cherezov *et al.*, 2009 ▸; Smith & Desgreniers, 2009 ▸; Aishima *et al.*, 2010 ▸; Bowler *et al.*, 2010 ▸; Hilgart *et al.*, 2011 ▸; Stepanov *et al.*, 2011 ▸; Scarborough *et al.*, 2017 ▸). However, they are usually optimized for micro-beams with very high photon fluxes, employ non-standard equipment or diffractometer geometries, and rely on an unobscured X-ray path to the sample. Centering approaches based on the shadowing of the primary X-ray beam (Sowa, 1994 ▸; Angel *et al.*, 2000 ▸; Budzianowski & Katrusiak, 2004 ▸; Dawson *et al.*, 2004 ▸) by the gasket can be utilized employing a laboratory source. Yet, they may require the reduction of generator power to avoid detector damage and only lead to an alignment of the gasket hole and not the sample position on the goniometer center.

For LT/HP experiments at the HTD2, we have developed a simple and robust diffraction-based centering procedure as an alternative to the concepts described above. It relies on crystal-position scans along different translation vectors and a subsequent search for the maximum of the recorded diffracted intensity. A controlled crystal translation is enabled by the motorized cryostat holder described in Section 2.1. Its axes *x*_cryo_, *y*_cryo_ and *z*_cryo_ [indicated in Figs. 3 ▸(*a*) and 5 ▸] rotate with the spindle axis φ and therefore allow for a positioning of the crystal into the goniometer center.

The optimal crystal alignment is determined by centering scans along three independent directions perpendicular to the X-ray beam, *i.e.* usually two translation scans perpendicular to the rotation axis (φ) at two φ positions differing by ∼90° and one translation scan parallel to the rotation axis [see Fig. 6 ▸(*a*)]. We note that non-perpendicular crystal translations with respect to the beam result in artificially broadened position–intensity profiles and hamper a precise determination of the goniometer center position. To simplify the task of defining three such scans irrespective of the current ω and χ settings, translation vectors in *HTD2CONTROL* can be specified in a fixed laboratory axis system in micrometres. Necessary conversions to the required positions of the cryostat holder axes *x*_cryo_, *y*_cryo_ and *z*_cryo_ are performed automatically. Notably, resulting translation scans may also involve combinations of movements along *x*_cryo_, *y*_cryo_ and *z*_cryo_ to ensure orthogonality of the scanning direction with respect to the X-ray beam.

Slight modifications to the aforementioned centering procedure are required if the investigated sample is contained in a pressure cell featuring an opening angle <90° (*e.g.* the T-DAC). In this case, the rotation angle difference between the two centering scans perpendicular to the rotation axis is necessarily smaller than 90°. By employing combined translations along the cryostat holder axes both centering scans can still be performed at right angles to the X-ray beam (*e.g.* by a simultaneous movement of the cryostat holder axes *x*_cryo_ and *y*_cryo_). But due to the rotation angle difference of <90° between the centering scans the according translation vectors cannot be linearly independent. Hence, the goniometer center position perpendicular to the rotation axis must be determined by an iterative procedure [see Fig. 6 ▸(*b*)]. The goniometer center position parallel to the rotation axis, however, can still be obtained by a single centering scan (usually by a stepwise crystal translation along the cryostat holder axis *z*_cryo_).

**Figure 5:**
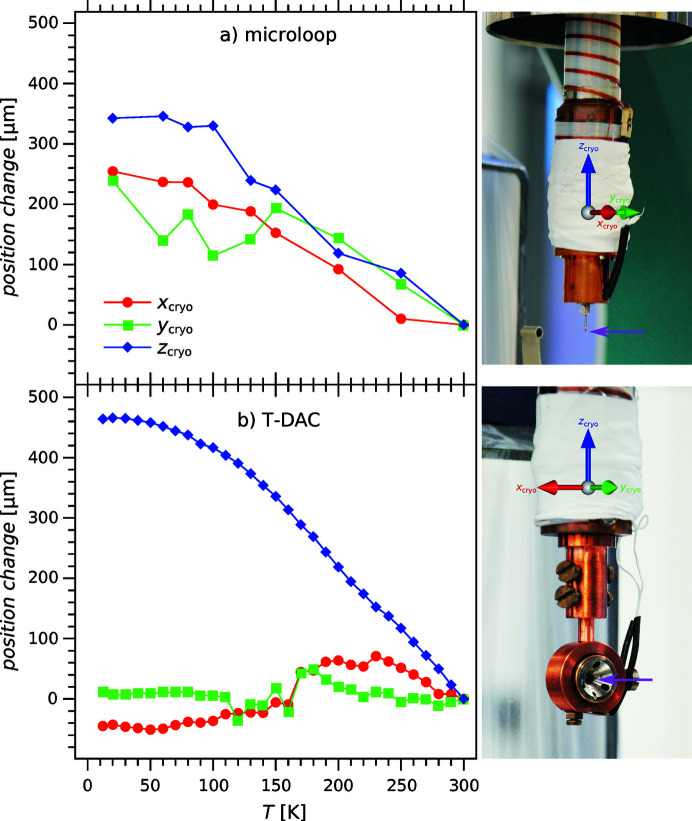
Temperature-dependent changes of the crystal position in directions parallel to the sample mount axis, *i.e.* the *z*_cryo_ axis (blue); and perpendicular to it, *i.e.* along the *x*_cryo_ (red) and *y*_cryo_ axes (green). Panels (*a*) and (*b*) illustrate the cases of ambient-pressure LT experiments with the sample (position indicated by a magenta arrow) mounted on a MiTeGen microloop and of HP/LT experiments with the sample placed inside a T-DAC, respectively. For clarity, the heat and radiation shields of the cryostat have been removed to show the approximate position of the sample.

**Figure 6:**
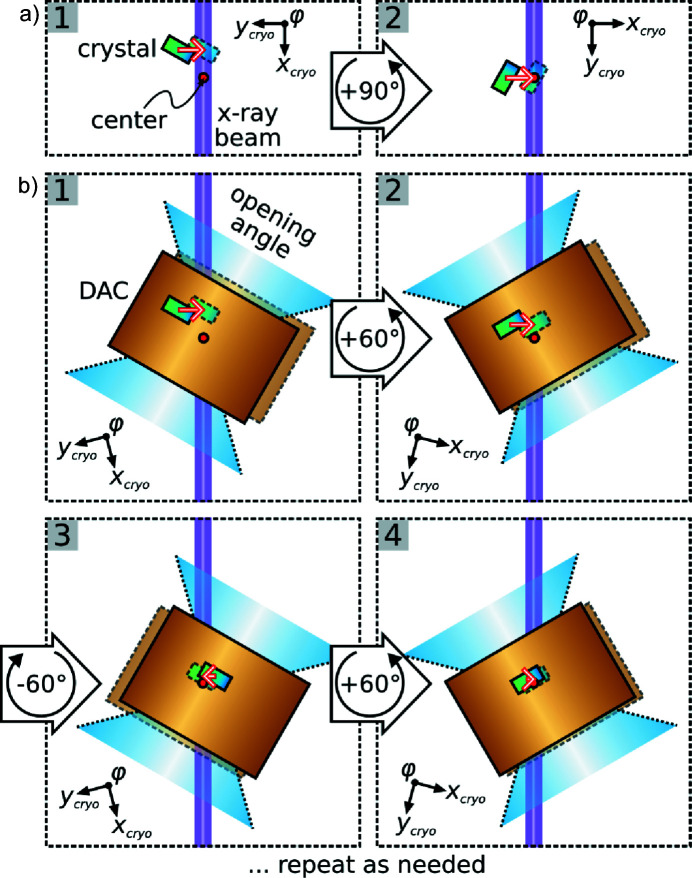
Schematic crystal centering perpendicular to the rotation axis in the case of (*a*) a free-standing sample and (*b*) a sample inside a pressure cell with an opening angle <90°.

If the crystal shifts out of the goniometer center are small, the diffraction-based centering procedure can be fully automated. Thereby, an additional narrow-ranged φ scan to search for crystal reflections is performed before each translation scan. *HTD2CONTROL* is able to differentiate between crystal and parasitic/background scattering by means of various methods (*e.g.* using masks or an orientation matrix). Such an automatic crystal re-centering is essential for temperature scans over wide temperature ranges without user intervention. An application example is the tracking of superstructure reflection intensities for the transition metal carbide Sc_3_CoC_4_ over temperature ranges from ∼20 to 300 K at different applied pressures (Langmann *et al.*, 2021 ▸). Another application may be the temperature-dependent study of lattice parameters, yet with limited precision in comparison with dedicated instruments (Gałdecka, 2011 ▸; Parrish *et al.*, 2011 ▸; Toby, 2019 ▸); see the supporting information for further details. Both types of XRD experiments hinge on repetitive re-centering, as the thermal contraction/expansion of the sample mount gradually moves the crystal out of the X-ray beam. This is illustrated by Fig. 5 ▸(*a*) for a sample mounted on a MiTeGen microloop, and by Fig. 5 ▸(*b*) for a sample placed inside a T-DAC.

### 3.2. Background handling

To reach the lowest possible temperatures by means of conduction cooling with a helium cryostat, two beryllium domes serving as vacuum and radiation shields have to be placed around the sample. Scattering of the primary beam by the texturized polycrystalline beryllium of the domes leads to a background with speckled Debye–Scherrer rings (Coome *et al.*, 2012 ▸; McMonagle & Probert, 2019 ▸) [see Fig. 2 ▸(*c*)]. As scattering by the inner and outer beryllium dome occurs at different distances to the detector plane, the resulting rings do not overlay in the collected diffraction images.

In the case of ambient-pressure LT experiments we reduce this background by subtracting images that only contain the beryllium scattering contributions. However, these have to be re-collected for every LT experiment by performing each φ scan twice: once with the sample centered in the X-ray beam, and once with the sample translated out of the center by a piezo nanopositioner (see Section 2.1). The underlying reason for this complex procedure lies in the grain structure of the polycrystalline beryllium walls, which leads to a dependence of the intensity pattern of the Debye–Scherrer rings on the current goniometer settings. Moreover, thermal contraction or expansion of the beryllium domes results in a temperature dependency of the location of the parasitic scattering contributions.

LT XRD data for Sc_3_CoC_4_ [*T* = 11 K; taken from Langmann *et al.* (2021 ▸)] show the success of the background-subtraction strategy. Between 82 and 150 K, the compound undergoes a structural phase transition from an orthorhombic high-temperature (HT; space group *Immm*) to a monoclinic LT (space group *C*2/*m*) phase accompanied by systematic pseudo-merohedral twinning (Vogt *et al.*, 2009 ▸; Eickerling *et al.*, 2013 ▸; Langmann *et al.*, 2020 ▸, 2021 ▸). XRD images for the needle-like single crystals of Sc_3_CoC_4_ span a large intensity range with very weak domain-specific superstructure reflections and relatively strong composite main reflections. A comparison of structural refinement results obtained using *JANA2006* (Petříček *et al.*, 2014 ▸) after data integration using the *EVAL14* data integration routine (Duisenberg *et al.*, 2003 ▸) and data reduction using *TWINABS* (Krause *et al.*, 2015 ▸) for the same XRD experiment without and with background subtraction is given in Table 1 ▸ (for more details see the supporting information). To ensure consistency, identical parameters have been used in the data integration and reduction processes.

It is evident that 693 reflections are automatically discarded by the data integration or reduction routines, when no background subtraction procedure is applied. Even more importantly, the reflection intensities obtained after background subtraction are of superior quality, as demonstrated by the strong decrease of all *R*_int_ values specified in Table 1 ▸. Weak domain-specific superstructure reflections profit most strongly, with *R*_int_ reductions of 53.9 (domain 1) and 53.5% (domain 2) compared with 20.0% for strong composite reflections. These improvements allow us to obtain a more accurate model of the subtle structural changes between the HT and LT phases of Sc_3_CoC_4_. Namely, all refined *R* values and the GooF of the final structural model drop by more than 20%. The estimated standard deviations of the derived bond lengths are also significantly reduced by more than 60% on average.

Yet, in the case of combined LT/HP studies the subtraction of parasitic beryllium scattering from the vacuum and radiation shields is impossible, since the high mass of the employed T-DAC prevents the use of the presently installed piezo nanopositioner inside the Displex. As an alternative, frame regions subject to shadowing (*e.g.* by the T-DAC body) or strong parasitic scattering (*e.g.* Debye–Scherrer rings due to beryllium scattering or reflections from the diamonds in the T-DAC) may be masked (Dawson *et al.*, 2004 ▸; Katrusiak, 2004 ▸; Macchi, 2012 ▸; McMahon *et al.*, 2013 ▸; Hejny & Minkov, 2015 ▸). This functionality is provided by a number of non-commercial stand-alone software tools (Kuhs *et al.*, 1996 ▸; Allan *et al.*, 2000 ▸; Angel, 2004 ▸; Dawson *et al.*, 2004 ▸; Parsons, 2004 ▸; Casati *et al.*, 2007 ▸; Angel & Gonzalez-Platas, 2013 ▸; Dera *et al.*, 2013 ▸) or commercial diffractometer software packages like *APEX* (Bruker, 2012 ▸), *CrysAlisPRO* (Agilent, 2014 ▸) and *X-Area* (Stoe & Cie, 2002 ▸). For convenient masking of XRD frames prior to data integration with the software *EVAL* (Duisenberg *et al.*, 2003 ▸; Schreurs *et al.*, 2010 ▸) we have developed a versatile Python program providing a graphical user interface. Masking of frame pixels is accomplished by assignment of a negative intensity value (−1.0) to mark them for removal in the subsequent data integration process. A range of the masking capabilities of the program has already been employed successfully in the determination of the LT/P structure of Sc_3_CoC_4_ (Langmann *et al.*, 2021 ▸).

### 3.3. Measurements under ambient conditions

Performing XRD experiments suitable for charge-density analysis is one of the most demanding types of measurement with respect to data quality. In order to evaluate the data quality achievable on the native instrument, we collected data for an α-boron single crystal at room temperature with a resolution of sin(θ)λ^−1^ ≤ 1.67 Å^−1^. This rather high data resolution exceeds the minimum required sin(θ)_max_λ^−1^ for standard charge-density studies by far and even goes beyond our previously published high-quality experimental study on α-boron (Fischer *et al.*, 2021 ▸) which serves as a benchmark for the present evaluation. After integration with *EVAL15* (Schreurs *et al.*, 2010 ▸) and data reduction with *SADABS* (Krause *et al.*, 2015 ▸), the newly collected XRD data were used first to refine an independent atom model (IAM) and then to refine a Hansen–Coppens multipolar model (HCM) (Hansen & Coppens, 1978 ▸) using *JANA2006* (Petříček *et al.*, 2014 ▸). For all refinements, only data with *I* > 3σ(*I*) were used. Table 2 ▸ summarizes salient experimental parameters and refinement results for the data sets from this study and from Fischer *et al.* (2021 ▸); more details are available in the supporting information.

The crystal structure of α-boron can be described as a slightly deformed cubic close packaging of B_12_ icosahedra formed by two independent atoms in the asymmetric unit, termed B_p_ and B_e_. While B_p_ atoms form strong intericosahedral (2c2e)-*exo*-bonds, B_e_ atoms establish somewhat weaker intericosahedral (3c2e)-*exo*-bonds connecting three icosahedra. At the HCM level the *exo*-B_p_–B_p_ and *exo*-B_e_–B_e_ bond lengths are 1.67170 (11) and 2.01316 (13) Å, respectively. This is slightly longer than the respective bond lengths at temperatures of 90 K [1.66879 (14) and 2.00945 (19) Å; Fischer *et al.*, 2021 ▸] and 100 K [1.6676 (4) and 2.0105 (4) Å; Nishibori *et al.*, 2015 ▸].

The analysis of the experimentally obtained charge density distribution employing Bader’s quantum theory of atoms in molecules (Bader, 1994 ▸) reveals a charge transfer of 0.09*e* from B_p_ atoms to B_e_ atoms, in excellent agreement with high-quality density functional theory (DFT) calculations (0.08*e*) and previous experimental studies by Fischer *et al.* (2021 ▸) (0.17*e*) and Mondal *et al.* (2013 ▸) (0.07*e*). Importantly, the previously reported 3D network of critical points is fully recovered.

**Figure 7:**
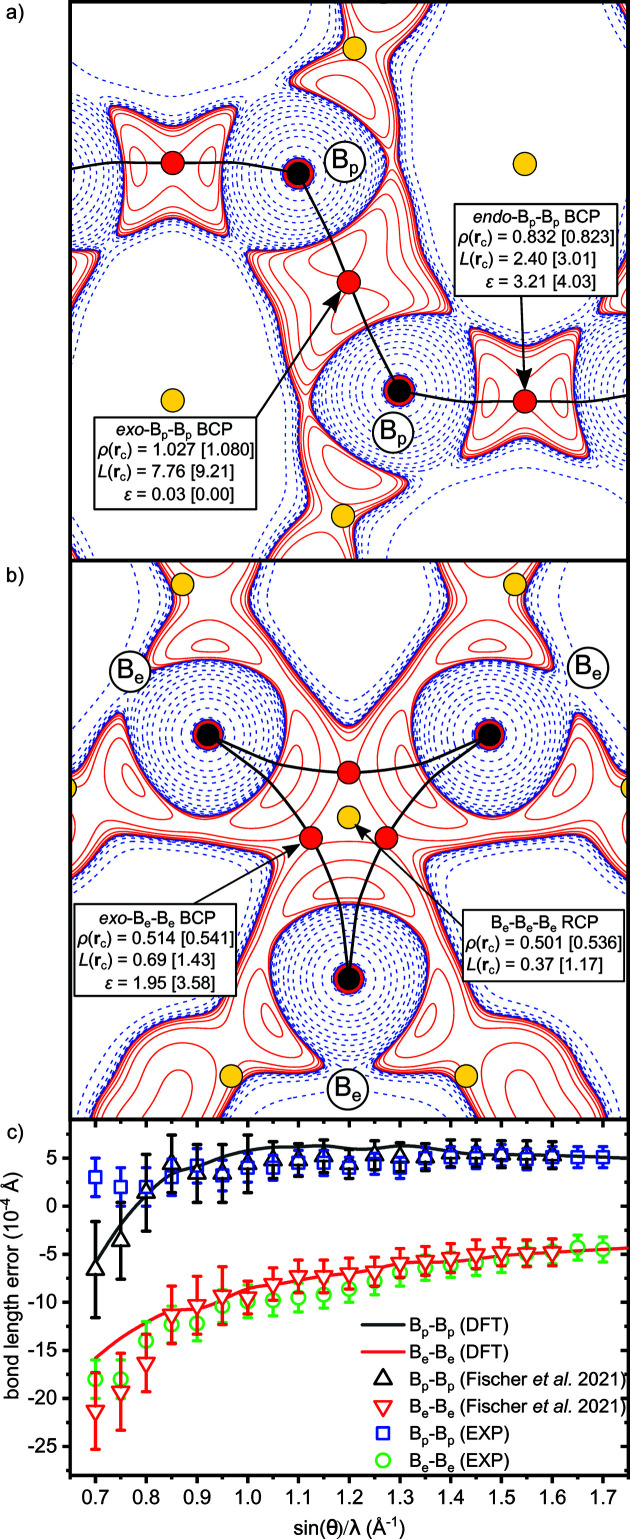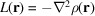 maps of (*a*) the intericosahedral (2*c*2*e*)-B_p_–B_p_ bond and (*b*) the (3*c*2*e*)-B_e_–B_e_–B_e_ bond in α-boron based on the experimental HCM refinement. Positive (solid red) and negative (dashed blue) contour lines are shown at levels of ±2 × 10^*n*^, ±4 × 10^*n*^ and ±8 × 10^*n*^ e Å^−5^ with *n* = ±3, ±2, ±1, 0. ρ(**r**_c_) and *L*(**r**_c_) values for selected bonds are given in units of e Å^−3^ and e Å^−5^, respectively. Reference values from DFT calculations by Fischer *et al.* (2021 ▸) are indicated by square brackets; (*c*) resolution dependence of the bond length errors due to ‘core asphericity shifts’ within the HCM. DFT data for referencing are taken from Fischer *et al.* (2021 ▸) and data points at the highest resolution are matched. Experimental data from Fischer *et al.* (2021 ▸) are shown for comparison.

*L*(**r**) maps (Laplacian of the electron density) of the two selected bonds as described above are shown in Figs. 7 ▸(*a*) and 7 ▸(*b*). As demonstrated by a detailed table in the supporting information, the values of ρ(**r**_c_), *L*(**r**_c_) and ε at the indicated bond- and ring-critical points are in very good agreement with both DFT-derived values and previous experimental studies (Mondal *et al.*, 2013 ▸; Fischer *et al.*, 2021 ▸). We note, however, a slight tendency of the ρ(**r**_c_) values from the present HTD2 data set to be systematically smaller (0.03–0.05 e Å^−3^) than in earlier experimental studies.

In a final step of our brief analysis we focus on the precision of the refined bond lengths. The currently used HCM is not flexible enough to capture the known dipolar core polarization effects in α-boron that lead to ‘core asphericity shifts’ and cause systematic bond length errors (Fischer *et al.*, 2021 ▸). Still, the resolution dependence of the bond length error [Fig. 7 ▸(*c*); referenced to a value derived from DFT calculations at the maximum experimental resolution of 1.67 Å^−1^; for details see Fischer *et al.* (2021 ▸)] is in very good agreement with our previously published study, especially regarding the *exo*-B_e_–B_e_ bond. Although being out of the scope of the present work, this finding confirms the suitability of the data set even for advanced core-density refinement techniques. These allow for a correction of bond-length errors due to ‘core asphericity shifts’ that amount to 4–6 × 10^−1^ Å at sin(θ_max_)λ^−1^ = 1.67 Å^−1^, a significant deviation with respect to the precision of the herein determined bond lengths. Thus, the good match between our results and previous state-of-the-art charge-density studies demonstrates a working interplay between the hardware and software components of the HTD2.

### 3.4. Measurements under LT/HP conditions

It has been shown that the complex carbide Sc_3_CoC_4_ becomes a volume superconductor at a transition temperature *T*_c_ of 4.5 K under only modest hydrostatic pressures (*p* ≥ 0.5 GPa) (Wang *et al.*, 2016 ▸; Langmann *et al.*, 2021 ▸). At the same time, it was observed that application of pressure at LT only leads to subtle structural rearrangements compared with the LT-phase model at ambient pressure and 11 K (space group *C*2/*m*) (Vogt *et al.*, 2009 ▸; Eickerling *et al.*, 2013 ▸; Langmann *et al.*, 2021 ▸). Yet, previously published structural data were collected at ∼4 GPa and 37 K and therefore still above the *T*_c_ of the compound (Langmann *et al.*, 2021 ▸). This makes structural investigations under simultaneous LT/HP conditions in the bulk-superconducting regime of Sc_3_CoC_4_ (*T* ≤ 4.5 K; *p* ≥ 0.5 GPa) appealing.

Hence, using our LT/HP setup we performed XRD experiments (i) on bulk-superconducting Sc_3_CoC_4_ at ∼3.3 GPa and 2.0 (3) K; and (ii) on normal-conducting Sc_3_CoC_4_ at ∼3.3 GPa and 6.7 (1) K. For this purpose, a single crystal of the compound (dimensions 73 × 98 × 147 µm^3^) was placed inside the pressure chamber of the T-DAC and put under hydrostatic pressure using Daphne 7575 (Murata & Aoki, 2016 ▸) as a pressure-transmitting medium. Pressure determination using the ruby fluorescence method (Piermarini *et al.*, 1975 ▸; Datchi *et al.*, 2007 ▸; Shen *et al.*, 2020 ▸; Kantor, 2021 ▸) could only be performed at room temperature, so that the final pressures at LT may deviate from the specified values due to thermal contraction of the pressure cell components. LT conditions were realized by mounting the pressure cell on (i) the cooling stage of the helium-flow or (ii) the closed-cycle helium cryostat. Both cryostats were operated with inner and outer beryllium heat and radiation shields (sample chamber vacuum ∼10^−6^ mbar). In the case of the helium-flow cryostat, the duration of the experiment was limited to ∼24 h by the supply of liquid helium in the Dewar vessel, and continuous pumping with a rotary vane pump was necessary to reach the specified temperature of 2.0 (3) K. After diffraction-based crystal centering (see Section 3.1), a total of 5 and 20 φ scans at different goniometer settings were taken at (i) 2.0 (3) and (ii) 6.7 (1) K, respectively (exposure time 100 s; see run lists in the supporting information).

Due to pressure-induced de-twinning the reflections in both LT/HP data sets could be indexed by a single orientation matrix. Data integration was performed using *EVAL14* (Duisenberg *et al.*, 2003 ▸), and *SADABS* (Krause *et al.*, 2015 ▸) was employed for scaling and absorption correction. Frame regions affected by shadowing or parasitic scattering were excluded from the integration process by masking (see Section 3.2). On the basis of the obtained reflection intensities, structural models featuring isotropic atomic displacement parameters for all atoms in the unit cell were refined using *JANA2006* ▸ (Petříček *et al.*, 2014 ▸); model parameters are available in the supporting information.

**Figure 8:**
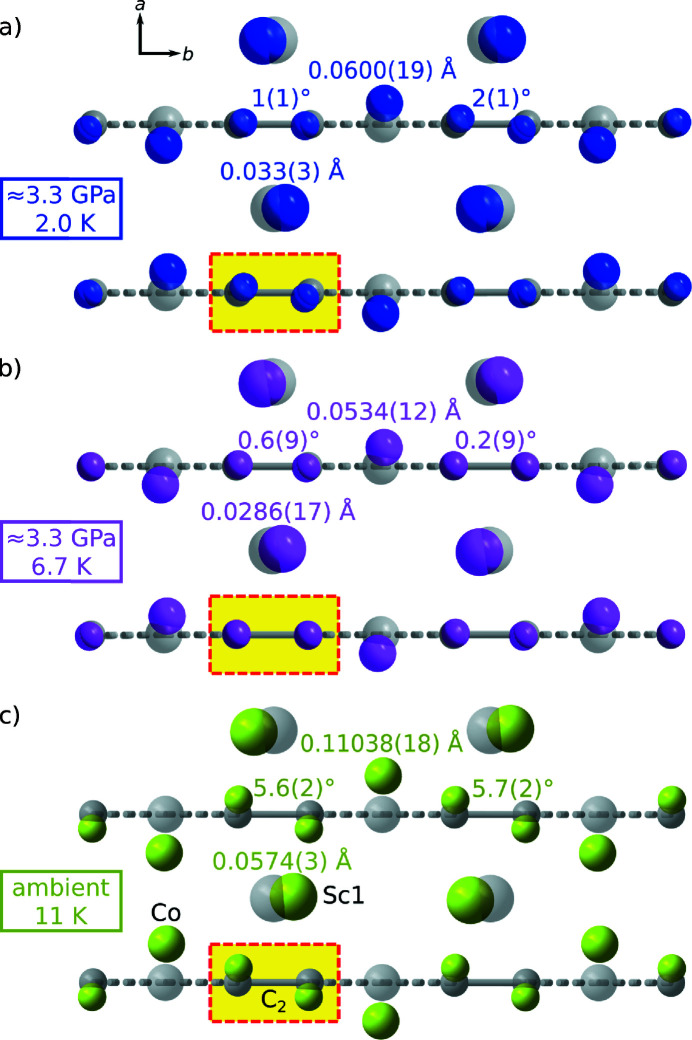
Ball-and-stick overlays of the refined atomic positions within a layered building unit of Sc_3_CoC_4_ under ambient conditions (gray, semi-transparent spheres) (Eickerling *et al.*, 2013 ▸) and after cooling to temperatures of (*a*) 2.0 (3) K and (*b*) 6.7 (1) K with an estimated applied pressure of 3.3 GPa (colored, non-transparent spheres). For reference, the structural changes on cooling to 11 (1) K under ambient-pressure conditions are depicted in (*c*) (Langmann *et al.*, 2021 ▸). The specified coordinate axes refer to the unit cell of the HT phase of Sc_3_CoC_4_. All atomic displacements away from the HT-phase model have been magnified by a factor of seven in the drawings. The respective refined values of atomic displacements and rotation angles are given with their threefold estimated standard deviations.

Figs. 8 ▸(*a*) and 8 ▸(*b*) illustrate the shifts obtained for the Co, Sc1 and C atoms away from their HT-phase positions (Eickerling *et al.*, 2013 ▸) (displacements magnified sevenfold). Relative to the HT-phase model, only the Co and Sc atoms are displaced significantly at 2.0 (3) [Fig. 8 ▸(*a*)] and 6.7 (1) K [Fig. 8 ▸(*b*)], while the C_2_ moieties remain close to their room-temperature positions. This is consistent with the previously published results at ∼4 GPa and 37 K (Langmann *et al.*, 2021 ▸). In the LT-phase structure at ambient pressure [Fig. 8 ▸(*c*)], by contrast, the C_2_ moieties are rotated by ∼6° about the *c* axis of the HT phase unit cell (Eickerling *et al.*, 2013 ▸; Langmann *et al.*, 2021 ▸). Taking into account the close resemblance of the atomic arrangements at 2.0 (3) and 6.7 (1) K, a strong structural reorganization during the superconducting transition of Sc_3_CoC_4_ can be excluded. Pressure-induced changes to the LT-phase structure are already present in the normal-conducting regime and remain stable on cooling below *T*_c_. Yet, these changes, *i.e.* a suppression of the rotation of the C_2_ moieties about the *c* axis (HT-phase unit cell), may be the prerequisite for Sc_3_CoC_4_ to enter a bulk-superconducting state under hydrostatic pressure conditions.

## 4. Conclusions and outlook

To conclude, we have provided a description of the setup and operation of the newly commissioned single-crystal X-ray diffractometer HTD2. It enables combined LT/HP diffraction studies under in-laboratory conditions. We could successfully collect high-resolution XRD data up to a reciprocal-space resolution of 1.67 Å^−1^ under ambient conditions and XRD data under simultaneous LT (2 K) and HP conditions (3.3 GPa). A comprehensive operating software package has been developed which controls the complex interplay of the diffractometer and the various cooling and HP devices and ensures a facile and automated operation during *T*/*p*-dependent diffraction experiments. This renders the HTD2 a versatile instrument in the mapping of structural control parameters of physical phenomena like superconductivity, the formation of charge-density waves or the onset of ferroelectricity.

## Supplementary Material

Crystal structure: contains datablock(s) global, Sc3CoC4_2K_3.3GPa, Sc3CoC4_6.7K_3.3GPa. DOI: 10.1107/S160057672200766X/iu5026sup1.cif

Structure factors: contains datablock(s) Sc3CoC4_2K_3.3GPa. DOI: 10.1107/S160057672200766X/iu5026sup2.hkl

Structure factors: contains datablock(s) Sc3CoC4_6.7K_3.3GPa. DOI: 10.1107/S160057672200766X/iu5026sup3.hkl

Details on high-resolution XRD experiments, low-temperature ambient-pressure XRD experiments, low-temperature/high-pressure XRD experiments, reproducibility of lattice parameters under low-temperature/ambient-pressure conditions. DOI: 10.1107/S160057672200766X/iu5026sup4.pdf

CCDC references: 2193017, 2193018

## Figures and Tables

**Table:** 

Empirical formula	Sc_3_CoC_4_		
Temperature (K)	11 (1)		
Crystal system, space group, *Z*	Monoclinic, *C*2/*m*, 4		
*a* (Å)	5.53630 (10)		
*b* (Å)	12.0210 (2)		
*c* (Å)	5.53640 (10)		
β (°)	104.8070 (10)		
*V* (Å^3^)	356.222 (11)		
Crystal size (µm^3^)	40 × 51 × 290		
Wavelength (Å)	0.56087		
Absorption coefficient (mm^−1^)	5.016		
Absorption correction type	Multi-scan		

**Table:** 

	Background subtraction		
	Without	With	Change (%)
Available reflections			
Domain 1	2861	3099	+8.3
Domain 2	2860	3100	+8.4
Composite	2306	2521	+9.3
Total	8027	8720	+8.6

*R*_int_			
Domain 1	0.0858	0.0396	−53.9
Domain 2	0.0891	0.0414	−53.5
Composite	0.0115	0.0092	−20.0
Total	0.0198	0.0123	−37.9

*R* [*I* > 2σ(*I*)]	0.0284	0.0220	−22.5
*wR* [*I* > 2σ(*I*)]	0.0635	0.0414	−34.8
*R*	0.0357	0.0271	−24.1
*wR*	0.0647	0.0424	−34.5
GooF (*F* ^2^)	1.66	1.27	−23.5

Largest peak/hole (e Å^−3^)	2.00/−2.05	1.97/−2.18	−1.5/+6.3

Bond length precision (Å)			
Co—C	0.004	0.0011	−63.3
C—C	0.009	0.002	−66.7

**Table 2:** Crystal data and details of the α-boron data set measured on the HTD2

	This study	Fischer *et al.* (2021 ▸)
Empirical formula	B	B
Temperature (K)	Room	90 (2)
Crystal system, space group, *Z*	Trigonal, *R* 3 *m*, 36	Trigonal, *R* 3 *m*, 36
*a* (Å)	4.9168 (1)	4.9085 (2)
*c* (Å)	12.5927 (3)	12.5697 (5)
*V* (Å^3^)	263.642 (12)	262.27 (2)

Crystal size (µm^3^)	75 × 139 × 140	75 × 139 × 140
Wavelength (Å)	0.56087	0.56087
Absorption coefficient (mm^−1^)	0.065	0.066
Absorption correction type	Numerical	Numerical
*F*(000)	180	180
ρ_calc_ (g cm^−3^)	2.451	2.464
Index ranges	−16 ≤ *h* ≤ 14	−15 ≤ *h* ≤ 13
	−14 ≤ *k* ≤ 16	−15 ≤ *k* ≤ 15
	−38 ≤ *l* ≤ 41	−38 ≤ *l* ≤ 39
2θ range (°)	7.66 < 2θ < 138.66	7.67 < 2θ < 124.70
Completeness (%)	98	99
Measured reflections	15740	23888
Unique reflections [*I* > 3σ(*I*)]	1282 (1094)	1093 (969)
*R*_int_, *R*_σ_	0.0213, 0.0089	0.0304, 0.0098

*R*_1_ (IAM)	0.0162	0.0173
*wR*_1_ (IAM)	0.0268	0.0283
GooF on *F* (IAM)	1.61	1.93
Largest peak/hole (IAM) (e Å^−3^)	+0.37/−0.20	+0.43/−0.20

*R*_1_ (HCM)	0.0105	0.0109
*wR*_1_ (HCM)	0.0149	0.0134
GooF on *F* (HCM)	0.91	0.93
Largest peak/hole (HCM) (e Å^−3^)	+0.17/−0.12	+0.18/−0.14

## Footnotes

1 Excellent overview articles covering general aspects of diffractometry under variable-temperature and HP conditions have been published by Peterson & Yang (2000 ▸), Miletich *et al.* (2000 ▸) and Angel *et al.* (2000 ▸).
