# Supplementary material for: HTD2: a single-crystal X-ray diffractometer for combined high-pressure/low-temperature experiments at laboratory scale
Source: J Appl Crystallogr. 2022 Sep 28;55(Pt 5):1255–66. doi: 10.1107/S160057672200766X (PMC9533757; doi:10.1107/S160057672200766X)
Supplement: Supplementary file 4 [file j-55-01255-sup4.pdf]

JOURNAL OF APPLIED CRYSTALLOGRAPHY

Volume 55 (2022)

Supporting information for article:

HTD2 – a single-crystal X-ray diffractometer for combined high-pressure/low-temperature experiments at laboratory scale

Andreas Fischer, Jan Langmann, Marcel Vöst, Georg Eickerling and Wolfgang Scherer

Supporting Information for: HTD2 - a single-crystal x-ray diffractometer for combined high-pressure / low-temperature experiments at lab scale

Andreas Fischer,[1] Jan Langmann,[1] Marcel Vöst,[1]
Georg Eickerling,[1,*] and Wolfgang Scherer[1,†]

[1] *CPM, Institut für Physik, Universität Augsburg, 86159 Augsburg, Germany*

(Dated: July 4, 2022)

CONTENTS

I. High-resolution XRD experiments 3
A. Investigated sample 3
B. Run list 4
C. Structural data 5
D. List of critical points of the electron density 7

II. Low-temperature / ambient-pressure XRD experiments 10
A. Investigated sample 10
B. Run list 11
C. Structural data 12

III. Low-temperature / high-pressure XRD experiments 15
A. Investigated sample and preparation of pressure cell 15
B. Run lists 16
C. Structural data 18

IV. Reproducibility of lattice parameters under low-temperature / ambient-pressure conditions 20
A. Run list 20
B. Result 21

References 22

I. HIGH-RESOLUTION XRD EXPERIMENTS

A. Investigated sample

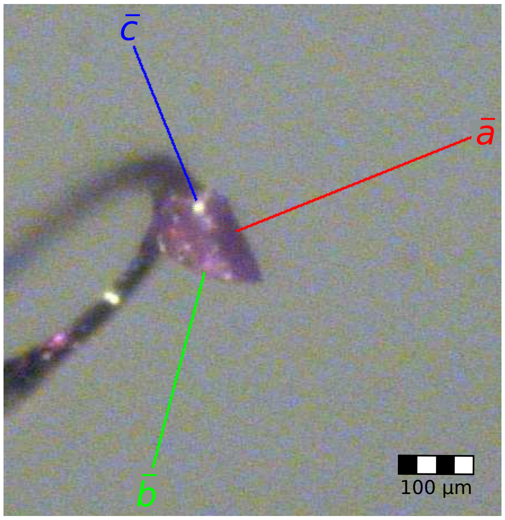

FIG. S1. Photographic image of the α-boron single crystal (synthesized according to literature methods[1]) used in the high-resolution x-ray diffraction experiment at ambient conditions. Crystal axes a, b and c referring to the trigonal unit cell are indicated by colored lines.

B. Run list

#	φ [°]	$\Delta\varphi$ [°]	$\Sigma\Delta\varphi$ [°]	Δt [s]	χ [°]	ω [°]	2θ [°]	Δ [mm]
1	-90	0.5	180	20	180	0	0	50
2	-90	0.5	180	20	150	0	0	50
3	-90	0.5	180	20	210	0	0	50
4	-90	0.5	180	50	180	34	34	50
5	-90	0.5	180	50	150	34	34	50
6	-90	0.5	180	50	210	34	34	50
7	-90	0.5	180	100	210	68	68	50
8	-90	0.5	180	100	180	68	68	50
9	-90	0.5	180	100	150	68	68	50
10	-90	0.5	180	240	180	-63.5	102	50
11	-90	0.5	180	240	210	-63.5	102	50
12	-90	0.5	180	240	150	-63.5	102	50

TABLE S1. Parameters of the φ scans for the α-boron single crystal (Fig. S1) at ambient conditions. (φ: scan starting angle; $\Delta\varphi$: scanned angle increment per frame; $\Sigma\Delta\varphi$: scanning range; Δt: exposure time per frame; Δ: detector to sample distance).

C. Structural data

unit cell dimensions	$a = 4.9168(1)$ Å<br>$c = 12.5927(3)$ Å<br>$V = 263.642(12)$ Å^3
calculated density	2.451 g·cm^{-3}
crystal size	75×139×140 µm^3
wave length	0.56087 Å
transm. ratio (max/min)	0.996 / 0.994
absorption coefficient	0.065 mm^{-1}
$F(000)$	180
2θ range	7.66° to 138.66°
range in hkl	-16/14, -14/16, -38/41
total no. reflections	15740
independent reflections	1282 ($R_{\mathrm{int}} = 0.0213$)
reflections with $I \geq 3\sigma(I)$	1094
data / parameters	1094 / 47
goodness-of-fit on F	0.91
R indices $[I \geq 3\sigma(I)]$	$R = 0.0105$<br>$wR = 0.0149$
extinction coefficient	1.6(2) (Becker & Coppens Type-II)
largest diff. peak and hole	+0.17 / -0.12 e·Å^{-3}

TABLE S2. Crystal data and structure refinement for a high-resolution single-crystal x-ray diffraction experiment on α-boron (Fig. S1) at ambient conditions.

$scale$	2.483(4)		ρ_{iso}	1.6(2)	
	B_p	B_e		B_p	B_e
x	0.23776(16)	0.196852(8)	P_{11+}	0.009(13)	-0.109(16)
y	0.11888	0.393704	P_{11-}	0.062(13)	-0.016(10)
z	0.108689(5)	0.024288(5)	P_{20}	-0.060(10)	0.021(10)
U_{11} [Å^2]	0.00324(2)	0.003947(17)	P_{22+}	-0.002(12)	-0.010(11)
U_{22} [Å^2]	0.003814(18)	0.00346(2)	P_{22-}	-0.103(10)	-0.010(9)
U_{33} [Å^2]	0.003109(18)	0.003780(18)	P_{31+}	0.052(11)	-0.092(14)
U_{12} [Å^2]	0.001620	0.001732	P_{31-}	-0.112(14)	-0.033(12)
U_{13} [Å^2]	-0.000375(11)	-0.000030(5)	P_{33+}	0.161(16)	0.149(18)
U_{23} [Å^2]	-0.000188	-0.000060	P_{33-}	0.019(10)	0.065(11)
U_{eq} [Å^2]	0.003451(13)	0.003784(13)	P_{40}	-0.008(12)	-0.023(18)
P_v	2.93(3)	3.07(3)	P_{42+}	-0.027(15)	0.011(18)
κ	0.985(9)	0.971(8)	P_{42-}	0.007(19)	0.016(22)
κ'	1.00(3)	0.96(4)	P_{44+}	0.016(16)	0.022(16)
			P_{44-}	0.009(12)	-0.047(13)

TABLE S3: HC model parameters refined against high-resolution XRD data ($(\sin\theta/\lambda)_{\max} \leq$ 1.67 Å^{-1}) collected on the α-boron single crystal (Fig. S1) at ambient conditions.

D. List of critical points of the electron density

#	study	type	m	$\rho(\mathbf{r}_c)$	$L(\mathbf{r}_c)$	ϵ	λ_3	location description
1	-	$(3,-3)$	6	-	-	-	-	B_p^a
2	-	$(3,-3)$	6	-	-	-	-	B_e^a
3	this study	$(3,-1)$	3	1.027	7.76	0.03	2.01	B_p^a-B_p^b (exo)
	Fischer[1]			1.079	9.40	0.05	0.69	
	Mondal[2]			1.104	9.57	-	-	
	DFT[1]			1.080	9.21	0.00	1.69	
4	this study	$(3,-1)$	6	0.832	2.40	3.21	2.15	B_p^a-B_p^c (endo)
	Fischer[1]			0.866	3.12	6.96	1.09	
	Mondal[2]			0.820	2.26	-	-	
	DFT[1]			0.823	3.01	4.03	1.33	
5	this study	$(3,-1)$	6	0.787	2.21	1.99	2.24	B_e^a-B_e^d (endo)
	Fischer[1]			0.817	3.02	2.31	1.32	
	Mondal[2]			0.804	2.47	-	-	
	DFT[1]			0.796	2.87	2.70	1.57	
6	this study	$(3,-1)$	6	0.715	1.30	6.07	2.14	B_p^a-B_e^e (endo)
	Fischer[1]			0.756	2.58	4.41	1.01	
	Mondal[2]			0.764	1.95	-	-	
	DFT[1]			0.768	2.60	3.45	1.45	
7	this study	$(3,-1)$	12	0.744	1.65	2.45	2.27	B_p^a-B_e^a (endo)
	Fischer[1]			0.756	1.93	3.93	1.44	
	Mondal[2]			0.745	1.39	-	-	
	DFT[1]			0.764	2.39	3.93	1.50	
8	this study	$(3,-1)$	6	0.514	0.69	1.95	2.07	B_e^a-B_e^f (endo)
	Fischer[1]			0.545	1.65	5.11	1.07	
	Mondal[2]			0.561	1.24	-	-	
	DFT[1]			0.541	1.43	3.58	1.18	

#	study	type	m	$\rho(\mathbf{r}_c)$	$L(\mathbf{r}_c)$	ϵ	λ_3	location description
9	this study	$(3,+1)$	2	0.817	1.60	-	-	B_p^a-B_p^c-B_p^h (endo)
	Fischer[1]			0.863	2.76	-	-	
	Mondal[2]			0.795	1.15	-	-	
	DFT[1]			0.807	2.15	-	-	
10	this study	$(3,+1)$	6	0.695	0.12	-	-	B_p^a-B_p^h-B_e^a (endo)
	Fischer[1]			0.731	0.67	-	-	
	Mondal[2]			0.704	1.96	-	-	
	DFT[1]			0.732	1.14	-	-	
11	this study	$(3,+1)$	12	0.694	0.41	-	-	B_p^a-B_e^a-B_e^e (endo)
	Fischer[1]			0.727	0.98	-	-	
	Mondal[2]			0.716	4.32	-	-	
	DFT[1]			0.728	1.19	-	-	
12	this study	$(3,+1)$	2	0.501	0.37	-	-	B_e^a-B_e^f-B_e^g (endo)
	Fischer[1]			0.543	1.53	-	-	
	Mondal[2]			0.557	1.06	-	-	
	DFT[1]			0.536	1.17	-	-	
13	this study	$(3,+1)$	3	0.269	–2.00	-	-	B_e^a-B_e^d-B_e^f-B_e^i (endo)
	Fischer[1]			0.277	–2.02	-	-	
	Mondal[2]			0.239	–2.10	-	-	
	DFT[1]			0.259	–1.88	-	-	
14	this study	$(3,+1)$	6	0.103	–0.98	-	-	side surfaces of tetrahedra formed by B_{12} icosahedra
	Fischer[1]			0.102	–1.10	-	-	
	DFT[1]			0.088	–1.05	-	-	

#	study	type	m	$\rho(\mathbf{r}_c)$	$L(\mathbf{r}_c)$	ϵ	λ_3	location description
15	this study	$(3,+3)$	1	0.086	–2.27	-	-	center of B_{12} icosahedron
	Fischer[1]			0.079	–2.71	-	-	
	DFT[1]			0.116	–2.05	-	-	
16	this study	$(3,+3)$	2	0.066	–0.78	-	-	tetrahedral void of B_{12} icosahedra
	Fischer[1]			0.059	–0.97	-	-	
	DFT[1]			0.059	–0.83	-	-	
17	this study	$(3,+3)$	1	0.035	–0.27	-	-	octahedral void of B_{12} icosahedra
	Fischer[1]			0.028	–0.31	-	-	
	DFT[1]			0.021	–0.26	-	-	

TABLE S4: Complete list of critical points in the topology of the electron density of α-boron as obtained from refinements of the x-ray diffraction data in this study with an HCM. For comparison, results from HCM refinements by Fischer *et al.*[1] and Mondal *et al.*[2] as well from DFT calculations[1] are shown. Values of $\rho(\mathbf{r}_c)$ are given in units of $e{\cdot}Å^{-3}$, values of $L(\mathbf{r}_c)$ and λ_3 are given in $e{\cdot}Å^{-5}$. m denotes the multiplicity of a critical point, while ϵ refers to the bond ellipticity for $(3,-1)$-type critical points. ^{a} x, y, z; ^{b} $-x+\frac{2}{3}, -x+y+\frac{1}{3}, -z+\frac{1}{3}$; ^{c} $-x+y, -x, z$; ^{d} $-x, -x+y, -z$; ^{e} $y, x, -z$; ^{f} $-x+y, -x+1, z$; ^{g} $-y+1, x-y+1, z$; ^{h} $-y, x-y, z$; ^{i} $x-y, -y+1, -z$.

II. LOW-TEMPERATURE / AMBIENT-PRESSURE XRD EXPERIMENTS

A. Investigated sample

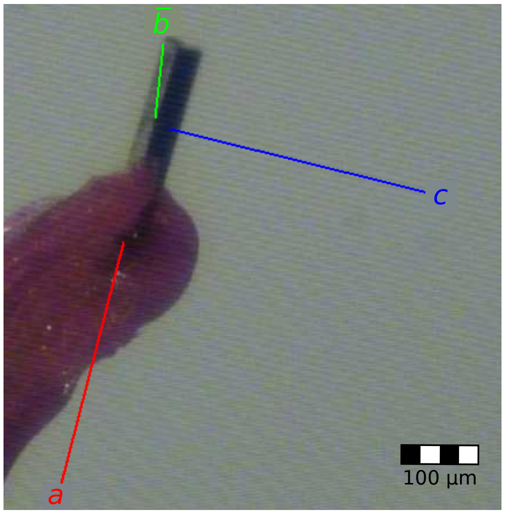

FIG. S2. Photographic image of the Sc_3CoC_4 single crystal (synthesized according to literature methods[3]) used in the x-ray diffraction experiment at ambient pressure and 11(1) K. Crystal axes a, b and c referring to the orthorhombic high-temperature phase unit cell are indicated by colored lines.

B. Run list

#	bgr.	φ [°]	$\Delta\varphi$ [°]	$\Sigma\Delta\varphi$ [°]	Δt [s]	χ [°]	$\omega/2\theta$ [°]	Δ [mm]
1	no	-90	0.5	180	120	150	0	70
2	yes	-90	0.5	180	120	150	0	70
3	no	-90	0.5	180	120	210	0	70
4	yes	-90	0.5	180	120	210	0	70
5	no	-90	0.5	180	120	150	20	70
6	yes	-90	0.5	180	120	150	20	70
7	no	-90	0.5	180	120	210	20	70
8	yes	-90	0.5	180	120	210	20	70
9	no	-90	0.5	180	120	150	40	70
10	yes	-90	0.5	180	120	150	40	70
11	no	-90	0.5	180	120	210	40	70
12	yes	-90	0.5	180	120	210	40	70

TABLE S5. Parameters of the φ scans for the Sc_3CoC_4 single crystal (Fig. S2) at ambient pressure and 11(1) K. (bgr.: scan is a background scan with the crystal translated out of the x-ray beam; φ: scan starting angle; $\Delta\varphi$: scanned angle increment per frame; $\Sigma\Delta\varphi$: scanning range; Δt: exposure time per frame; Δ: detector to sample distance).

C. Structural data

background	not subtracted	subtracted
unit cell dimensions	$a = 5.53630(10)$ Å<br>$b = 12.0210(2)$ Å<br>$c = 5.53640(10)$ Å<br>$\beta = 104.8070(10)°$<br>$V = 356.222(11)$ Å^3	
calculated density	4.5095 g·cm^{-3}	
crystal size	40×51×290 µm^3	
wave length	0.56087 Å	
transm. ratio (max/min)	0.747 / 0.643	0.747 / 0.686
absorption coefficient	5.016 mm^{-1}	
$F(000)$	456	
θ range	3° to 36°	
range in hkl	-11/11, -25/25, -11/11	
total no. reflections	8027	8720
independent reflections	2143 ($R_{\mathrm{int}} = 0.0198$)	2142 ($R_{\mathrm{int}} = 0.0123$)
reflections with $I \geq 2\sigma(I)$	1882	2007
data / parameters	2143 / 43	2142 / 43
goodness-of-fit on F^2	1.66	1.27
R indices $[I \geq 2\sigma(I)]$	$R = 0.0284$<br>$wR = 0.0635$	$R = 0.0220$<br>$wR = 0.0414$
R indices (all data)	$R = 0.0357$<br>$wR = 0.0647$	$R = 0.0271$<br>$wR = 0.0424$
extinction coefficient	0.052(2)<br>(SHELX model)	0.0461(14)<br>(SHELX model)
largest diff. peak and hole	2.00 / -2.05 e·Å^{-3}	1.97 / -2.18 e·Å^{-3}

TABLE S6. Crystal data and structure refinements for a single-crystal x-ray diffraction experiment on Sc_3CoC_4 (Fig. S2) at ambient pressure and 11(1) K without and with determination and subtraction of the parasitic scattering background from the beryllium heat and radiation shields (data set taken from Ref. 3).

		fractional atomic coordinates			U_{eq}
atom	bgr. sub.	x	y	z	[Å^2]
Co	no	0.26607(4)	0	0.26686(4)	0.00206(5)
	yes	0.26595(2)	0	0.26673(2)	0.00204(3)
Sc1	no	0.75575(4)	0	0.24266(4)	0.00214(10)
	yes	0.75582(3)	0	0.24273(3)	0.00207(6)
Sc2	no	0	0.187441(18)	0	0.00212(12)
	yes	0	0.187417(10)	0	0.00210(9)
Sc3	no	0	0.311565(18)	0.5	0.00207(12)
	yes	0	0.311540(10)	0.5	0.00210(9)
C1	no	0.4162(7)	0.12560(7)	0.0818(7)	0.0036(5)
	yes	0.4110(3)	0.12557(5)	0.0766(2)	0.0031(2)
C2	no	0.0838(7)	0.12495(7)	0.4180(7)	0.0036(5)
	yes	0.0889(3)	0.12487(5)	0.4233(2)	0.0030(2)

TABLE S7. Refined fractional atomic coordinates and mean-square atomic displacement parameters obtained from a single-crystal x-ray diffraction experiment on Sc_3CoC_4 (Fig. S2) at ambient pressure and 11(1) K without and with determination and subtraction of the parasitic scattering background from the beryllium heat and radiation shields (data set taken from Ref. 3).

		mean-square atomic displacement parameters [Å^2]					
atom	bgr. sub.	U_{11}	U_{22}	U_{33}	U_{12}	U_{13}	U_{23}
Co	no	0.00244(10)	0.00177(6)	0.00212(10)	*	0.00086(5)	*
	yes	0.00229(6)	0.00176(4)	0.00219(6)	*	0.00082(3)	*
Sc1	no	0.0023(2)	0.00197(8)	0.00217(19)	*	0.00063(7)	*
	yes	0.00223(12)	0.00202(5)	0.00195(12)	*	0.00053(5)	*
Sc2	no	0.0036(2)	0.00214(8)	0.0006(2)	*	0.00058(7)	*
	yes	0.00295(18)	0.00198(5)	0.00144(17)	*	0.00068(4)	*
Sc3	no	0.0038(2)	0.00190(8)	0.0005(2)	*	0.00063(7)	*
	yes	0.00295(18)	0.00196(5)	0.00147(17)	*	0.00071(4)	*
C1	no	0.0031(9)	0.0036(3)	0.0042(9)	-0.0004(8)	0.0011(3)	-0.0008(8)
	yes	0.0017(4)	0.00353(17)	0.0037(4)	0.0005(3)	0.00026(16)	0.0002(3)
C2	no	0.0034(9)	0.0035(3)	0.0044(9)	0.0007(8)	0.0016(3)	0.0009(8)
	yes	0.0019(4)	0.00337(17)	0.0037(4)	-0.0002(3)	0.00039(16)	0.0000(3)

TABLE S8. Refined mean-square atomic displacement parameters obtained from an ambient-pressure single-crystal x-ray diffraction experiment on Sc_3CoC_4 (Fig. S2) at 11(1) K without and with determination and subtraction of the parasitic scattering background from the beryllium heat and radiation shields (data set taken from Ref. 3). Parameters marked by an asterisk are forbidden by symmetry.

III. LOW-TEMPERATURE / HIGH-PRESSURE XRD EXPERIMENTS

A. Investigated sample and preparation of pressure cell

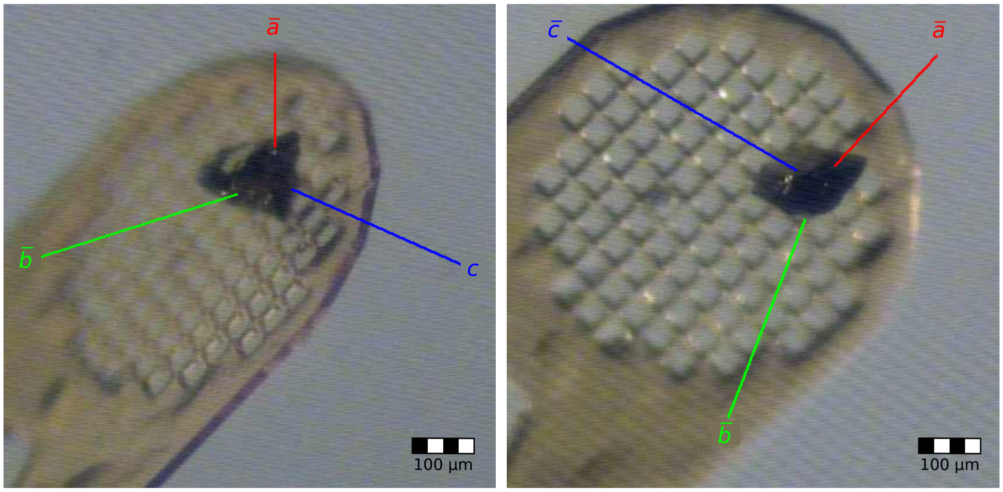

FIG. S3. Photographic images of the Sc_3CoC_4 single crystal (synthesized according to literature methods[3]) used in the low-temperature high-pressure x-ray diffraction experiments from different viewing angles. Crystal axes a, b and c referring to the orthorhombic high-temperature phase unit cell are indicated by colored lines.

The Sc_3CoC_4 single crystal (Fig. S3) got placed inside the pressure chamber of a Tozer-type DAC[4,5] (T-DAC, ALMAX EASYLAB) equipped with Boehler-Almax-type diamond anvils[6] of type Ia (culet size 600 µm). The pressure chamber is represented by a hole in the center of an initially pre-indented stainless steel gasket. The diameter of the hole was 255 µm, and the height of the pre-indentation was 108 µm. At the edge of the pressure chamber, two ruby spheres got positioned for pressure determination via the ruby-fluorescence method.[7–10] As a pressure transmitting medium Daphne 7575 got employed, providing hydrostatic conditions up to a maximum pressure between 3.9 GPa and 4 GPa at 295°C.[11] After adding the pressure transmitting medium into the pressure chamber the pressure got increased to 3.3 GPa. The ruby fluorescence measurements got executed at room temperature.

After relaxation of the pressure the T-DAC got mounted in a suited copper holder, which itself is either connected to the cold finger of the helium-flow cryostat (*i*) or the closed-cycle helium cryocooler (*ii*). In both cases, two beryllium domes got placed around the T-DAC, serving as vacuum and radiation shields. Depending on which cryostat got applied,

a minimum temperature of 2.0(3) K (*i*) or 6.7(1) K (*ii*) got reached. Further details about the pressure- and temperature-dependent x-ray diffraction measurements as well as the data analysis are provided in Tab. S9 to Tab. S12 and in the text of the main paper.

B. Run lists

#	φ [°]	$\Delta\varphi$ [°]	$\Sigma\Delta\varphi$ [°]	Δt [s]	χ [°]	$\omega/2\theta$ [°]	Δ [mm]
1	-30.5	0.5	65.0	100	180	0	60
2	-2.5	0.5	57.5	100	180	28	60
3	-58.5	0.5	65.5	100	180	-28	60
4	-44.5	0.5	65.5	100	180	-14	60
5	-16.5	0.5	35.5	100	180	14	60

TABLE S9. Parameters of the φ scans for the Sc_3CoC_4 single crystal (Fig. S3) at 3.3 GPa and 2.0(3) K. (φ: scan starting angle; $\Delta\varphi$: scanned angle increment per frame; $\Sigma\Delta\varphi$: scanning range; Δt: exposure time per frame; Δ: detector to sample distance).

#	φ [°]	$\Delta\varphi$ [°]	$\Sigma\Delta\varphi$ [°]	Δt [s]	χ [°]	$\omega/2\theta$ [°]	Δ [mm]
1	-31.5	0.5	66.0	100	180	0	60
2	-3.5	0.5	53.5	100	180	28	60
3	-17.5	0.5	66.0	100	180	14	60
4	-45.5	0.5	66.0	100	180	-14	60
5	-54.0	0.5	60.5	100	180	-28	60
6	-31.5	0.5	65.0	100	150	0	60
7	-2.0	0.5	54.5	100	150	28	60
8	-17.5	0.5	63.5	100	150	14	60
9	-42.5	0.5	64.5	100	150	-14	60
10	-55.5	0.5	63.0	100	150	-28	60
11	-31.5	0.5	65.5	100	210	0	60
12	-2.0	0.5	59.0	100	210	28	60
13	-17.5	0.5	63.0	100	210	14	60
14	-54.5	0.5	61.5	100	210	-28	60
15	-42.5	0.5	64.0	100	210	-14	60
16	-31.5	0.5	66.0	100	180	0	85
17	-3.5	0.5	58.0	100	180	28	85
18	-17.5	0.5	64.5	100	180	14	85
19	-51.5	0.5	58.5	100	180	-28	85
20	-45.5	0.5	66.5	100	180	-14	85

TABLE S10. Parameters of the φ scans for the Sc_3CoC_4 single crystal (Fig. S3) at 3.3 GPa and 6.7(1) K. (φ: scan starting angle; $\Delta\varphi$: scanned angle increment per frame; $\Sigma\Delta\varphi$: scanning range; Δt: exposure time per frame; Δ: detector to sample distance).

C. Structural data

T [K]	2.0(3)	6.7(1)
unit cell dimensions	$a = 5.5300(4)$ Å	$a = 5.5124(4)$ Å
	$b = 11.9606(15)$ Å	$b = 11.9341(14)$ Å
	$c = 5.5350(6)$ Å	$c = 5.5167(6)$ Å
	$\beta = 104.508(3)°$	$\beta = 104.413(3)°$
	$V = 354.42(6)$ Å^3	$V = 351.50(6)$ Å^3
calculated density	4.5324 g·cm^{-3}	4.5701 g·cm^{-3}
crystal size	73×98×147 µm^3	
wave length	0.56087 Å	
transm. ratio (max/min)	0.648 / 0.390	0.648 / 0.560
absorption coefficient	5.041 mm^{-1}	5.083 mm^{-1}
$F(000)$	456	
θ range	3° to 31°	
range in hkl	-7/7, -14/15, -6/6	-9/8, -15/16, -6/7
total no. reflections	679	2523
independent reflections	189 ($R_{\text{int}} = 0.0363$)	255 ($R_{\text{int}} = 0.0207$)
reflections with $I \geq 1.5\sigma(I)$	165	228
data / parameters	165 / 18	228 / 18
goodness-of-fit on F^2	2.26	3.33
R indices [$I \geq 1.5\sigma(I)$]	$R = 0.0583$	$R = 0.0615$
	$wR = 0.1427$	$wR = 0.1339$
R indices (all data)	$R = 0.0583$	$R = 0.0615$
	$wR = 0.1427$	$wR = 0.1339$
extinction coefficient	–	–
largest diff. peak and hole	0.92 / -0.90 e·Å^{-3}	2.32 / -2.13 e·Å^{-3}

TABLE S11. Crystal data and structure refinements for single-crystal x-ray diffraction experiments on Sc_3CoC_4 (Fig. S3) at a pressure of 3.3 GPa and temperatures of 2.0(3) K and 6.7(1) K.

atom	T [K]	fractional atomic coordinates x	y	z	U_{eq} [Å^2]
Co	2.0(3)	0.2582(2)	0	0.2595(3)	0.0010(5)
	6.7(1)	0.25683(13)	0	0.25897(18)	0.0021(4)
Sc1	2.0(3)	0.7534(3)	0	0.2460(4)	0.0006(6)
	6.7(1)	0.75256(17)	0	0.2460(2)	0.0021(4)
Sc2	2.0(3)	0	0.18819(19)	0	0.0009(5)
	6.7(1)	0	0.18784(12)	0	0.0018(4)
Sc3	2.0(3)	0	0.31127(19)	0.5	0.0006(5)
	6.7(1)	0	0.31155(12)	0.5	0.0018(4)
C1	2.0(3)	0.4180(16)	0.1240(7)	0.0808(17)	0.0029(12)
	6.7(1)	0.4170(10)	0.1243(5)	0.0817(12)	0.0039(8)
C2	2.0(3)	0.0836(16)	0.1235(7)	0.4210(17)	0.0029(12)
	6.7(1)	0.0832(10)	0.1238(5)	0.4173(12)	0.0039(8)

TABLE S12. Refined fractional atomic coordinates and mean-square atomic displacement parameters obtained from single-crystal x-ray diffraction experiments on Sc_3CoC_4 (Fig. S3) at a pressure of 3.3 GPa and temperatures of 2.0(3) K and 6.7(1) K.

IV. REPRODUCIBILITY OF LATTICE PARAMETERS UNDER LOW-TEMPERATURE / AMBIENT-PRESSURE CONDITIONS

A. Run list

#	bgr.	φ [°]	$\Delta\varphi$ [°]	$\Sigma\Delta\varphi$ [°]	Δt [s]	χ [°]	$\omega/2\theta$ [°]	Δ [mm]
1	no	-90	0.5	180	40	180	28	55
2	yes	-90	0.5	180	40	180	28	55

TABLE S13. Parameters of the repeated φ scans in a case study of the lattice parameter precision for an α-boron single crystal (Fig. S1) at ambient pressure and 11(1) K. (bgr.: scan is a background scan with the crystal translated out of the x-ray beam; φ: scan starting angle; $\Delta\varphi$: scanned angle increment per frame; $\Sigma\Delta\varphi$: scanning range; Δt: exposure time per frame; Δ: detector to sample distance).

B. Result

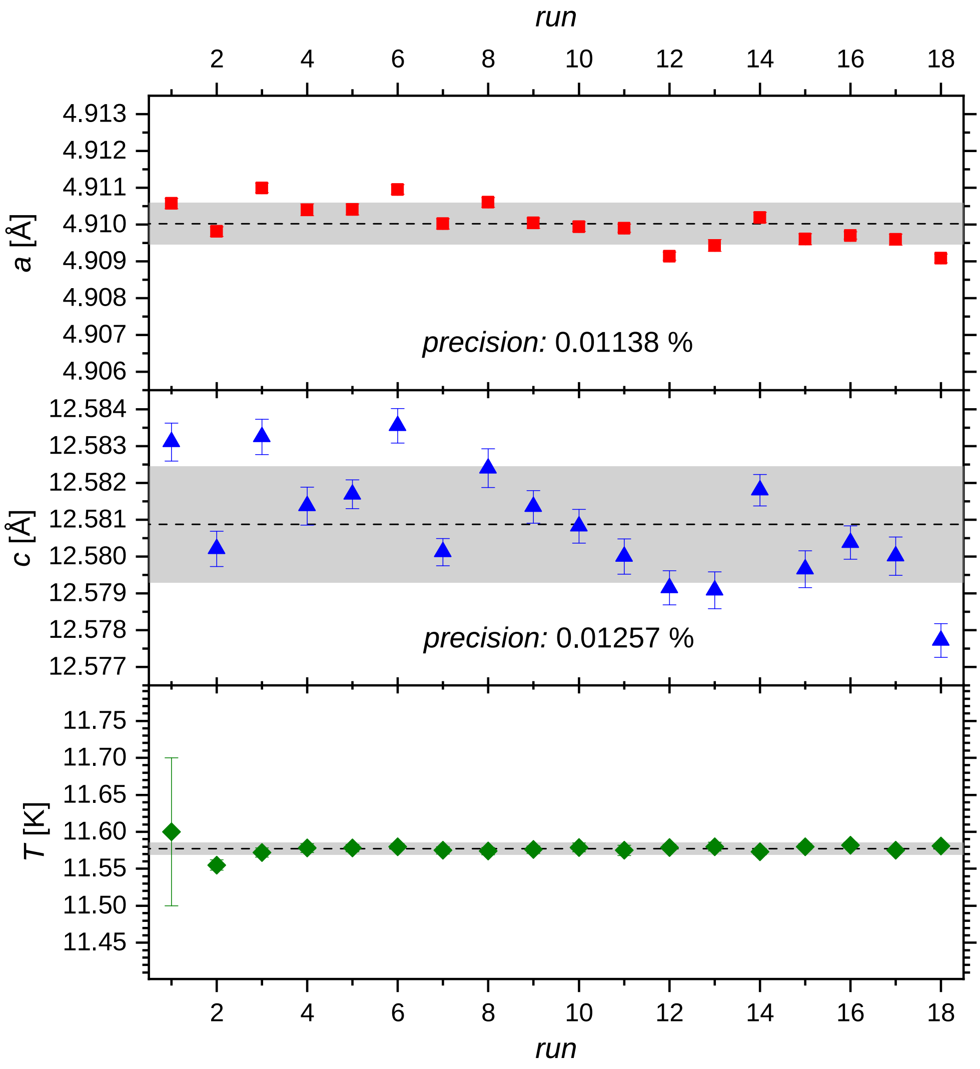

FIG. S4. Variation of refined lattice parameters and sample temperature for an α-boron single crystal (Fig. S1) kept at ambient pressure and an average temperature of 11.577(8) K upon repeated execution of the φ scan set in Tab. S13. The dashed line and the gray-shaded region mark the average value and standard deviation over all obtained lattice parameters and sample temperatures, respectively. We note that EVAL14[12] has been used for data integration and lattice parameter refinement.

* georg.eickerling@uni-a.de

† wolfgang.scherer@uni-a.de

[1] A. Fischer, G. Eickerling, and W. Scherer, Molecules **26**, 4270 (2021).

[2] S. Mondal, S. van Smaalen, G. Parakhonskiy, S. J. Prathapa, L. Noohinejad, E. Bykova, N. Dubrovinskaia, D. Chernyshov, and L. Dubrovinsky, Phys. Rev. B **88**, 024118 (2013).

[3] J. Langmann, M. Vöst, D. Schmitz, C. Haas, G. Eickerling, A. Jesche, M. Nicklas, A. Lanza, N. Casati, P. Macchi, and W. Scherer, Phys. Rev. B **103**, 184101 (2021).

[4] D. E. Graf, R. L. Stillwell, K. M. Purcell, and S. W. Tozer, High Press. Res. **31**, 533 (2011).

[5] ALMAX EASYLAB, "Product details Diacell® TozerDAC-Xray," (2021), [online; `www.almax-easylab.com/ProductDetails.aspx?PID=118`; accessed 7-December-2021].

[6] R. Boehler and K. De Hantsetters, High Press. Res. **24**, 391 (2004).

[7] G. J. Piermarini, S. Block, J. D. Barnett, and R. A. Forman, J. Appl. Phys. **46**, 2774 (1975).

[8] I. Kantor, "Fluorescense pressure calculation and thermocouple tools," (2021), [online; `kantor.50webs.com/ruby.htm`; accessed 30-November-2021].

[9] G. Shen, Y. Wang, A. Dewaele, C. Wu, D. E. Fratanduono, J. Eggert, S. Klotz, K. F. Dziubek, P. Loubeyre, O. V. Fat'yanov, P. D. Asimow, T. Mashimo, R. M. M. Wentzcovitch, and other members of the IPPS task group, High Press. Res. **40**, 299 (2020).

[10] F. Datchi, A. Dewaele, P. Loubeyre, R. Letoullec, Y. Le Godec, and B. Canny, High Press. Res. **27**, 447 (2007).

[11] K. Murata and S. Aoki, Rev. High Pressure Sci. Technol. **26**, 3 (2016).

[12] A. J. M. Duisenberg, L. M. J. Kroon-Batenburg, and A. M. M. Schreurs, J. Appl. Cryst. **36**, 220 (2003).
